# Robot-aided fN∙m torque sensing within an ultrawide dynamic range

**DOI:** 10.1038/s41378-020-00231-0

**Published:** 2021-01-04

**Authors:** Shudong Wang, Xueyong Wei, Haojian Lu, Ziming Ren, Zhuangde Jiang, Juan Ren, Zhan Yang, Lining Sun, Wanfeng Shang, Xinyu Wu, Yajing Shen

**Affiliations:** 1 State Key Laboratory for Manufacturing Systems Engineering, Xi’an Jiaotong University, Xi’an, 710049 China; 2 Mechanical and Biomedical Engineering Department, City University of Hong Kong, Hong Kong, SAR 999077 China; 3 Department of Mechatronics, Chang’an University, Xi’an, 710054 China; 4 Robotics and Microsystems Center, Soochow University, Suzhou, 215021 China; 5 Shenzhen Institutes of Advanced Technology, Chinese Academy of Sciences, Shenzhen, 518055 China; 6 Shenzhen Research Institute, City University of Hong Kong, Shenzhen, 440305 China

**Keywords:** Electrical and electronic engineering, Structural properties

## Abstract

In situ scanning electron microscope (SEM) characterization have enabled the stretching, compression, and bending of micro/nanomaterials and have greatly expanded our understanding of small-scale phenomena. However, as one of the fundamental approaches for material analytics, torsion tests at a small scale remain a major challenge due to the lack of an ultrahigh precise torque sensor and the delicate sample assembly strategy. Herein, we present a microelectromechanical resonant torque sensor with an ultrahigh resolution of up to 4.78 fN∙m within an ultrawide dynamic range of 123 dB. Moreover, we propose a nanorobotic system to realize the precise assembly of microscale specimens with nanoscale positioning accuracy and to conduct repeatable in situ pure torsion tests for the first time. As a demonstration, we characterized the mechanical properties of Si microbeams through torsion tests and found that these microbeams were five-fold stronger than their bulk counterparts. The proposed torsion characterization system pushes the limit of mechanical torsion tests, overcomes the deficiencies in current in situ characterization techniques, and expands our knowledge regarding the behavior of micro/nanomaterials at various loads, which is expected to have significant implications for the eventual development and implementation of materials science.

## Sensors: measuring torque

A microelectromechanical resonant sensor is proposed for small-scale torsion tests and demonstrated for the testing of silicon microbeams. It is important to understand the mechanical properties of small-scale materials used in MEMS and NEMS. Various systems have been reported for this under a range of loading conditions, but the application of torque to small-scale samples is particularly hard to measure. Here, a joint team led by Xueyong Wei from Xi’an Jiaotong University and Yajing Shen from City University of Hong Kong reports a MEMS-based resonator sensor that can measure bending and torsion over a wide dynamic range with a high force resolution. They demonstrate their system for the testing of silicon microbeams, which are found to be five times stronger than bulk silicon under pure torsion.

## Introduction

The scale-dependent mechanical properties of materials have been a fundamental issue in scientific and industrial applications. When the characteristic length of a material is on the micro or nanoscale, particularly in micro/nanoelectromechanical system (MEMS/NEMS), the scale effect becomes dominant and unavoidable in the analysis and design of materials and structural systems^1^. Therefore, a quantitative measurement of scale effects can help scientists comprehend the properties of micro/nanomaterials^2,3^ and provide guidance for the topology design of various devices^4–7^. As a fundamental approach for small-scale material analytics^8^, the torsion test plays an indispensable role in promoting an understanding of the world at the micro/nanoscale. For instance, peculiar phenomena, including grain boundary sliding^9^ and crystal twinning^10^, can be directly observed under torsion. In addition, various devices, including micromirrors^11^, MEMS gyroscopes^12^, microturbines^13^, and biomedical devices^14^, are subjected to torsional stress during operation, making torsional analysis an essential method for material evaluation.

An atomic force microscopy (AFM)-based system is a commonly used technique for nanocharacterization due to its highly precise positioning and force sensing ability. In addition to the conventional compression and bending test^15^, AFM has been successfully applied to the twisting test of nanomaterials, such as carbon nanotubes (CNTs), based on the optical beam deflection approach^16–18^. However, such a setup is not applicable to twist the specimen with a large angle due to the low manipulation flexibility of the AFM system. Moreover, it is also difficult to obtain high-resolution imaging information and the fracture features of the specimen in the test, which may limit the in-depth understanding of the material’s behavior. In contrast, in situ scanning electron microscopy (SEM) characterization can operate on micro/nano specimens in 3-D space and offers real nanometer resolution imaging ability. Currently, this technique is regarded as an effective method to study the scale effect of micro/nanomaterials^19^, and recent advances have enabled the in situ SEM tensile/bending testing of micro/nanomaterials^20,21^. However, torsion testing at a small scale remains a major challenge due to its requirements for high-resolution torque sensing, large manipulation flexibility, and accurate assembly capability^22,23^.

Undoubtedly, the force/torque sensor, as a key part of the torsion test, ultimately determines the testing performance. Force/torque sensors reported thus far are mostly based on the principles of the capacitance^24^, piezoresistance^25^, and electromagnetism^26^, which successfully enabled the mechanical analysis of materials with dimensions of tens or hundreds of microns but cannot satisfy the requirements for torsion tests at smaller scales. The handling and flexible operation of small specimens are additional major challenges that need to be addressed to carry out in situ SEM torsion testing^27^. Large-scale sophisticated equipment, such as a torsion balance, can hardly be arranged inside the narrow space of an SEM chamber^28^. In addition, limited by the inherent monocular vision of SEM, only a 2-D surface image can be generated; thus, the rotational operation raises many serious challenges, including accurately approaching the target location, the nondestructive 3-D assembly of micro/nanostructures, and canceling the off-center error in rotation.

Herein, we present a robot-aided in situ bending/torsion characterization system. A laboratory-built MEMS resonant sensor is developed to provide tactile sensing and force/torque measurements for the system. Compared with previously reported counterparts, the developed sensors can achieve four orders of magnitude improvement in resolution and an ultrawide dynamic range of 123 dB. Moreover, we introduce a multifunctional nanorobotics system for the assembly of small-scale specimens. Aided by exquisite tactile sensation and SEM vision, the system can perform mechanical tests, including bending and pure torsion, with various kinds of materials. Subtle interoperation strategies of the two nanorobots are carried out to reduce the off-center error of torsional manipulation and demonstrate repeatable loading-unloading tests. For the first time, we propose pure torsion tests for micron/submicron-scale specimens and find that Si microbeams are five-fold stronger than their bulk counterparts. The seamless integration of our robotic torsion test system provides a general and powerful solution for the in situ mechanical testing of micro/nanomaterials, thereby enabling a wide spectrum of applications.

## Results and discussion

### Development of a robot-aided in situ torsion characterization system

The robot-aided in situ torsion characterization system was constructed with a nanorobotics system and a self-developed microelectromechanical resonant torque sensor inside the SEM chamber, whereas a control system and a monitoring system were located outside the chamber. Taking advantage of the integrated manipulation system equipped with SEM visualization and tactile sensors, we performed complex microassembly under hybrid feedback and carried out bending/torsion tests on micron/submicron-scale specimens.

**Fig. 1:**
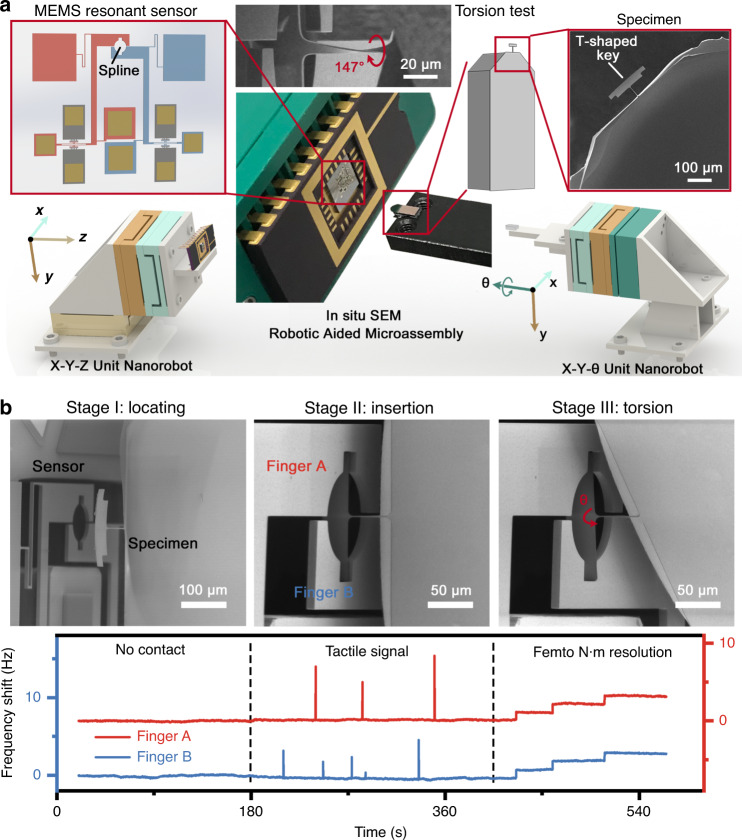
Development of a robot-aided in situ torsion characterization system. **a** Systematic diagram of the robot-aided in situ torsion characterization system. **b** Different roles of visual sensing and tactile sensing during the three stages of characterization

The nanorobotics system consists of two independent three degree-of-freedom (DoF) nanorobots, i.e., the X–Y–θ unit nanorobot (left) and the X–Y–Z unit nanorobot (right), as shown in Fig. 1a. The X–Y–θ robot was integrated with two linear positioners and a rotary positioner, whereas the X–Y–Z robot was composed of three orthogonal linear positioners. In the figure, the different colors indicate six nanorobots with diverse functions. The homemade cross-scale specimens and the MEMS sensor were rigidly fixed on the left and right separately. The nanorobotics system was mounted on a rotary platform inside the SEM chamber, which allows the SEM lens to observe the specimen from an oblique perspective, thereby roughly guiding the manipulation. All electrical instruments, including the nanoscale motion controller, vector network analyzer, digital current power sources, and computers, were arranged outside the SEM chamber (see Supplementary Fig. S1). Additionally, a specific vacuum flange was designed to realize the information interaction inside and outside the cavity.

Figure 1b indicates the guidance provided by the visual and tactile information during practical tasks. In the initial position (stage I), where the focus was far from the sensor plane, the distance between the two can be roughly estimated through the SEM micrograph, while the output of the MEMS sensor remained constant. During the insertion (stage II), the shadow effect^29^ in the micrograph was used to roughly identify the 3-D locomotion. Moreover, the quick response and high stability of the MEMS sensor allowed us to determine a touching signal through observation of an abnormal pulse. Upon completion of the microassembly (stage III), the MEMS sensor further served as a strain gauge that can measure the force/torque applied on the cross-scale specimen. With its femtonewton-meter resolution and submillinewton-meter measurement range, such a sensor is qualified for material analytics with different dimensions.

### Microelectromechanical resonant torque sensor with ultrahigh resolution and an ultrawide dynamic range

Mechanical tests for specimens with various dimensions pose a challenge for the performance of the torque sensors. The resolution, measurement range, and dynamic range should be optimized simultaneously to distinguish small force variations from the noise floor and to endure the relatively high stress produced when the specimen fails. Therefore, the sensor proposed in this paper was designed based on the resonance sensing principle instead of traditional capacitance or piezoresistive methods.

**Fig. 2:**
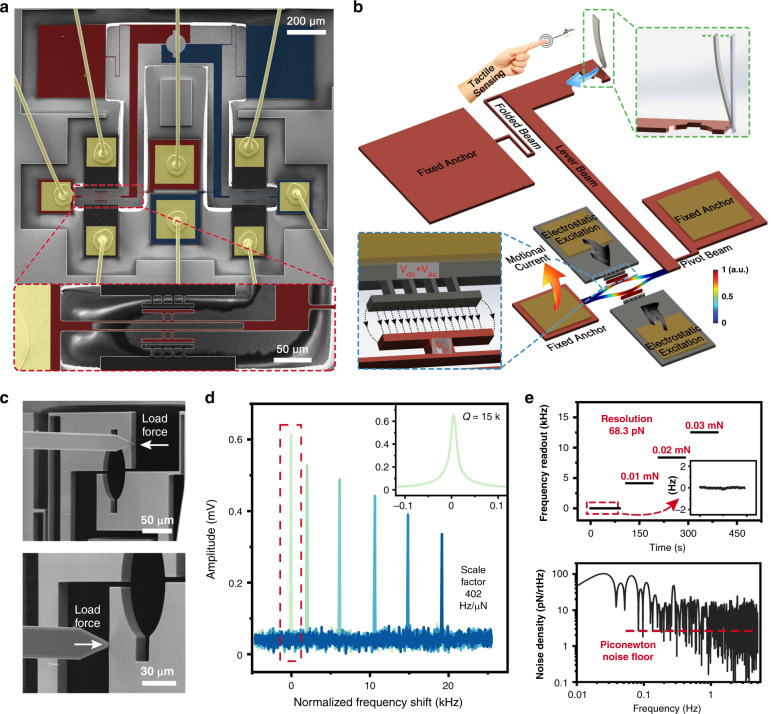
Design and calibration of the ultrahigh precise torque sensor. **a** False-colored SEM image of the sensor from the top view and detailed micrograph of the DETF (red dashed box). **b** Operation mechanism of the sensor and its calibration. **c** Micrograph of the calibration experiment. Two finger beams were calibrated separately. **d** Open-loop test results of DETF A using a vector network analyzer. **e** Real-time frequency output via self-excitation oscillation shows a low noise floor of approximately 1–10 pN/rt-Hz

The sensor was designed and optimized using the finite element method (COMSOL Multiphysics 5.4) and was fabricated through a commercially available silicon-on-insulator manufacturing process (MEMSCAP Inc., USA). The topological structure of the sensor was mainly integrated in the top Si layer, as shown in Fig. 2a. The sensor consists of two symmetrical parts: the released elements, i.e., the DETFs and the finger beams, and the fixed components, i.e., the anchors and the electrodes. A spline was etched in the middle of the finger beam, where the cross-scale specimen was to be inserted. When the material is bent or twisted, the reaction force will be applied on the splines, magnified, and transmitted to the DETF resonators. The characteristic frequency of the resonator will be changed according to the following formula^30^:

1 $$f = f_0\sqrt {1 + \lambda P}$$

where *f* is the frequency of the resonator, *f*_0_ is its natural frequency, *λ* is related to the dimensions of the resonator, and *P* is the axial force applied on the resonator.

The simulated scale factor of the sensor is 388 Hz/μN. However, due to the inherent error in MEMS manufacturing, the practical scale factor is different from the theoretical value, and thus, it is necessary to conduct calibration experiments. Figure 2b demonstrates the calibration experiment of the sensor. An atomic force microscopy (AFM) cantilever with a measured spring constant k = 1.93 ± 0.06 N/m (calibrated by a nanomanipulation approach^31^, in agreement with the nominal stiffness of 2 N/m) was installed on the X–Y–θ robot, and the sensor was mounted on the X–Y–Z robot. The whole nanorobotics system was fixed inside the SEM chamber with a 25-degree incline. The cantilever was manipulated to apply stress on the finger beam in its sensitive direction, as shown in Fig. 2c. Thus, a certain force will be applied:

2 $$F_{{\mathrm{Bending}}} = k_{{\mathrm{AFM}}} \cdot \left( {\Delta l_{Ro} - \frac{{n \cdot \Delta x}}{{sin\theta _s}}} \right)$$

where *F*_Bending_ is the bending force applied on the finger beam, *k*_AFM_ is the stiffness of the AFM cantilever, Δ*l*_*Ro*_ is the movement of the nanopositioner, *n* is the number of pixels that the finger beam has moved, Δ*x* is the distance of a single pixel, and *θ*_*s*_ is the rotation angle of the stage inside the SEM chamber.

At the same time, the electrodes on both sides of the DETF were charged with an alternating voltage $\left( {V_{{\mathrm{dc}}} + V_{{\mathrm{ac}}} = 100\,{\mathrm{V}} \pm 5\,{\mathrm{mV}}} \right)$, providing a frequency-sweep excitation force through the parallel plate. In this way, the DETF resonator can be excited to vibrate and generate a motional current through the parallel plate capacitance. A vector network analyzer was utilized to record the motional current and restore the resonant peak. The results of the frequency-sweep open-loop test are shown in Fig. 2d. The eigenfrequencies of DETF A and DETF B are approximately 338 kHz and 335 kHz, respectively. The Q-factor of the DETF resonator was approximately 15k, depending on the vacuum degree. As the axial force increased, the frequency of the resonance peak increased, and according to the relationship between the applied force and DETF frequency, the obtained scale factors of DETF A and DETF B are 402 Hz/μN and 385 Hz/μN, respectively.

In the closed-loop experiment, the DETF resonator was then embedded in a self-oscillation circuit (see Supplementary Fig. S2a). The circuit can track the characteristic frequency of the DETF and achieve a real-time frequency reading with a sampling rate of 10 Hz. Figure 2e shows the results of the closed-loop test, whose scale factor coincides with that of the open-loop test. The power spectral density analysis on the frequency of DETF A indicates a noise floor of approximately 1–10 pN/rt-Hz. In addition, the Allan deviation of its zero-bias output indicates that the theoretical force and torque resolution of the proposed sensors were 68.3 pN and 4.78 fN·m, respectively (see Supplementary Fig. S2b). Compared with existing sensors^22,24–26,32–34^, the proposed sensor has a ten thousand times higher resolution and a one hundred times wider dynamic range (Table 1), which allows it to measure the mechanical properties of the materials at the micron/submicron scale.

**Table 1:** Performance of reported force/torque sensors

Sensing mechanism	Force (N)		Torque (N·m)		Dynamic range (dB)
	Resolution	Range	Resolution	Range	
Capacitance^24^	1E−6	1.42E−4	1E−9	2.26E−6	73
Capacitance^32^	1.4E−6	1E−3	3.6E−9	2.6E−6	57
Piezoresistance^33^	1.3E−5	4E−3	1.1E−8	4E−6	51
Piezoresistance^25^	1E−5	1E−4	\	\	20
Optical^34^	4.5E−2	\	\	\	\
Capacitance^22^	1.9E−2	\	4.43E−3	\	\
Electromagnetism^26^	\	\	1E−9	1.6E−5	104
This work	6.8E−11	1E−4	4.78E−15	7E−9	123

### Fabrication of the specimens

The proposed cross-scale specimen was fabricated using a silicon-on-insulator wafer (see Supplementary Fig. S3). The silicon beams lie on the <110> or the <100> crystalline orientation. The processing flow is as follows. First, front-side photolithography (photoresist AZ6130, thickness of 2.5 μm) was performed to define the specimen pattern, and then inductively coupled plasma (ICP) etching was conducted to transfer the pattern to the top silicon layer. To obtain the suspended specimen, backside photolithography (photoresist AZ4620, thickness of 6.5 μm) was performed to define the backside window, followed by deep reactive ion etching (DRIE) of the handle silicon layer. There are two options for the last step. If the silicon dry etching method was used, then silicon dioxide samples can be obtained, and if wet etching through HF was applied, then silicon specimens can be obtained.

### Robot-aided microassembly of the specimens

Microassembly under SEM is a rapidly developing field that has a substantial influence on a series of industrial and research applications. However, a scanning electron microscope has only one lens; as a result, the 3-D relationship cannot be clearly outlined by the 2-D diagram. The 3-D position of the assembly cannot be fully reconstructed, resulting in a considerable assembly error. Therefore, we introduce a microassembly strategy based on the hybrid control method, i.e., visual feedback control and tactile feedback control. We utilized the interaction between the elements to eliminate the displacement of one dimension, turning the 3-D microassembly into a pseudo 2-D assembly.

The objects to be assembled in this work are a MEMS sensor and a cross-scale specimen, which were separately fixed on the left and right nanorobots. During installation, an inevitable installation error between the rotation center of the nanorobot and the axis of the cross-scale specimen exists, and thus, the prealignment of the robotic system is essential. The assembly process is divided into three steps: searching for the in situ torsion center, finding the target location, and insertion of the cross-scale specimen. To precisely implement the torsion test and the insertion process, the distance between the axis of the specimen and the rotational center of the robot must be eliminated. However, this operation is nearly impossible to achieve manually. Therefore, we developed a three-point alignment method^35^ (see Supplementary Fig. S4). The off-center error can be dramatically reduced after several alignment steps. However, a systematic error lasts in the end, which cannot be canceled through this operation. This error needs to be further eliminated by the interaction between the two nanorobots.

As mentioned above, positional information aliasing can be introduced when the 2-D SEM image guides the 3-D assembly. Therefore, we developed a target locating approach based on visual feedback and tactile feedback. In the initial position, large portions of the image were outside the focal plane except for the cross-scale specimen, which indicates a considerable distance between the specimen and the sensor plane. Next, the *z*-axis nanopositioner of the X–Y–θ robot was utilized to push the sensor forward until the two objects touched. Since only a few parts of the sensor, i.e., the two finger beams, were enabled with tactile sensing, the relationship between the two objects can only be estimated through visual feedback. When touching the sensor, backscattered electrons underneath the specimen were blocked, darkening the corresponding image area and creating a shadow on the sensor plane. After recording the touch-point image and referring to the sensor map in Fig. 2a, we can find the location and calculate Δ*x* and Δ*y* between the touch point and the target. Next, we let the *z*-axis nanopositioner retract a certain distance, and then, manipulators *x* and *y* move so that the target location can be approached. It should be noted that the position of the specimen in the world coordinates did not change during this process, as any unnecessary movement may lead to a superfluous off-center error. After repeating the above process several times, the specimen will find the spline position (see Supplementary Movie S1).

**Fig. 3:**
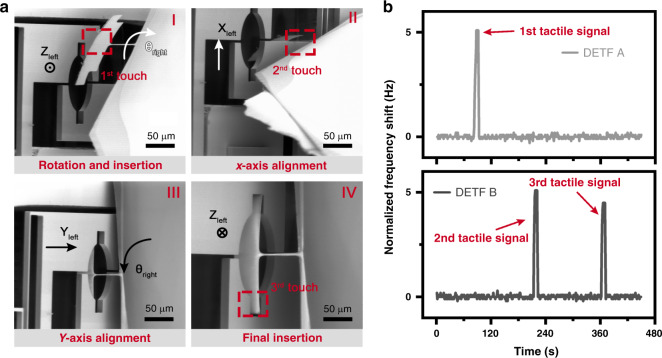
Microassembly of the specimen based on the hybrid control method. **a** Insertion processing guided by tactile feedback. **b** When the specimen and the finger beam interact, stress will be transmitted to the corresponding DETF, leading to a frequency shift of several Hertz

After that, the specimen needs to be inserted into the spline. As demonstrated in Fig. 3a, the field of view is parallel to the *x*-axis and perpendicular to the *y*-axis and *z*-axis, so the latter two axes cannot be distinguished clearly in the micrograph. Therefore, tactile feedback plays a crucial role during microassembly. Since the head of the specimen is wider than the spline, the specimen must be inserted into the spline through four steps, i.e., rotation and insertion through the blank area (I), *x*-axis alignment (II), *y*-axis alignment (III), and the final insertion (IV). During the assembly, a slight bump between the specimen and the finger beam was inevitable (red dashed boxes). When the bump occurs, due to the slow response and positional information aliasing of the visual feedback, it cannot be found until a large deformation occurs, which might damage the surface of the samples, leading to an erroneous testing result. However, by utilizing tactile feedback, we were able to efficiently detect the interaction through the frequency shift of the two DETFs and thus protect the fragile specimens from crashing into the sensor. As shown in Fig. 3b, when Δ*f* was greater than a certain value, e.g., 5 Hz, the system could determine that a bump had occurred, regardless of whether it could be detected in the SEM image. Afterward, the specimen was manipulated to retract and bypass obstacles as needed. After tactile feedback was utilized to guide the assembly, the deformation of the cross-scale specimen was significantly reduced.

### In situ bending and pure torsion characterization

**Fig. 4:**
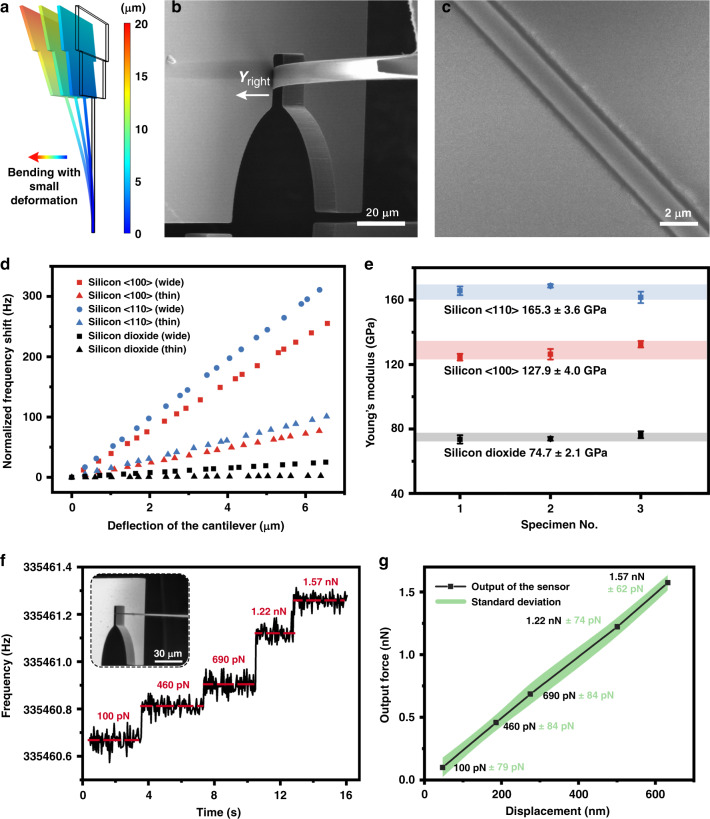
Bending test with ultrahigh force resolution. **a** Bending test with a small deformation in the finite element analysis simulation. **b** Micrograph of the bending test. **c** Zoomed-in SEM micrograph of the microbeam. **d** Testing results for various specimens. The different slopes show the different stresses applied to the material as it bends. **e** Young’s modulus measurement results of nine specimens. The small variance obtained from multiple tests of a single sample shows the high repeatability of the system. **f** Resolution demonstration through the bending test. **g** Displacement of the beam versus the output force of the sensor

Based on the abovementioned microassembly and prealignment strategy, a demonstration of in situ bending and pure torsion was carried out for several cross-scale specimens. Through the finite element method, we can find a linear relationship (*R*^2^ = 0.998) between the bending stress and deformation of the cross-scale specimen (when the deformation is smaller than 20 μm), as demonstrated in Fig. 4a. Therefore, during the experiment, we manipulated the free end of the specimen to touch the finger beam and then controlled the clamped end of the specimen to move forward with a step of approximately 150 nm and applied stress on the cantilever, as shown in Fig. 4b. The distance is precisely measured through the grating ruler integrated in the motion controller. Moreover, the frequency shift of the DETF was also recorded to deduce the stress on the specimen. Before the bending test, we performed dimension analysis on each specimen through the SEM micrograph, as demonstrated in Fig. 4c. The edges of the specimens can be extracted through the Canny operator, and the dimensions can be calculated through the averaging approach. Figure 4d shows the testing results of the frequency shift versus cantilever deformation for six different specimens. The force applied on the finger beams can be estimated by

3 $$F_{{\mathrm{Bending}}} = \frac{{\Delta f}}{{SF}}$$

where Δ*f* is the frequency shift of the corresponding DETF, and *SF* is its measured scale factor. In Fig. 4e, we conducted repeatability tests on different samples of silicon <100>, silicon <110> and silicon dioxide. The Young’s modulus can be easily obtained through Euler-Bernoulli theory. The results showed that the measurement error of a single sample was rather small, with an average standard deviation of 2.0%, while that of the different samples was less than 3.1%.

The practical resolution of the sensor was evaluated with a slender silicon dioxide cantilever. During the test, the specimen was bent slightly to induce a small force applied on the sensor, and the frequency output of DETF A was simultaneously recorded, as demonstrated in Fig. 4f. The results indicate that our sensor can easily distinguish a frequency shift of 0.1 Hz (248 pN according to the scale factor 402 Hz/μN). Figure 4g plots the calculated force versus the specimen displacement, which shows a perfect linear relationship (*R*^2^ = 0.9997) between the two. A uniform deviation of tens of pN within the measurement range of 0–1.57 nN was obtained, and the maximum standard deviation was found to be 84 pN, which is close to the theoretical resolution of 68.3 pN. According to the calibration experiment and the finite element simulation, the measurement range of such a sensor was approximately 0.1 mN, which indicates a wide dynamic range of 123 dB.

**Fig. 5:**
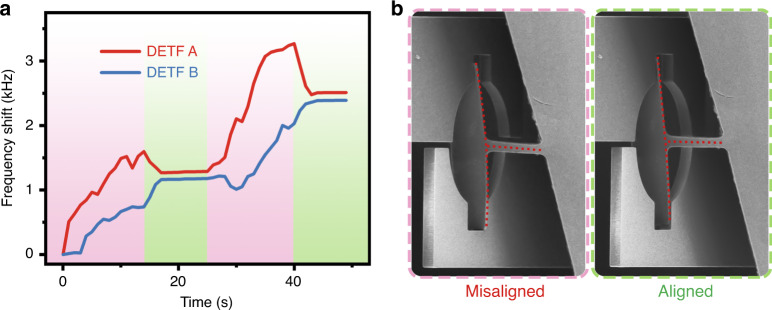
Pure torsion experiments under tactile feedback. **a** Rotation and realignment of the silicon specimen guided by the hybrid control method. **b** Micrograph of the specimen before (red) and after (green) realignment

We further performed a pure torsion test on a silicon cross-scale specimen (width = 8.35 μm, length = 50 μm, and thickness = 1.23 μm). During the rotation, the axis of the cross-scale specimen deviated from the center of the spline due to the existence of the off-center error, thereby introducing unnecessary tensile or bending stress. Although the torsion experiment can be performed under simple visual guidance, the measurement error will be relatively large. More accurate measurements can be performed under the real-time tactile feedback provided by the sensor. As shown in Fig. 5a, during the rotation, although the frequencies of both DETFs increased (the red area), the slopes were not constant and lacked consistency. This behavior is because the stresses applied on the two finger beams were different due to the off-center rotation. This problem can be solved through the realignment of the X–Y–Z unit nanorobot. When the normalized frequency shifts of DETFs A and B were proportional to their respective scale factors, it was proven that the off-center error was eliminated, as shown in the green region. SEM images were recorded during this process. Before realignment, the edges of the upper and lower finger beams were severely misaligned, and the axis of the specimen was also inclined (see the red part in Fig. 5b), while after alignment, these edges became ordered (green part). Through this approach, a pure torque can be applied on the cross-scale specimen.

**Fig. 6:**
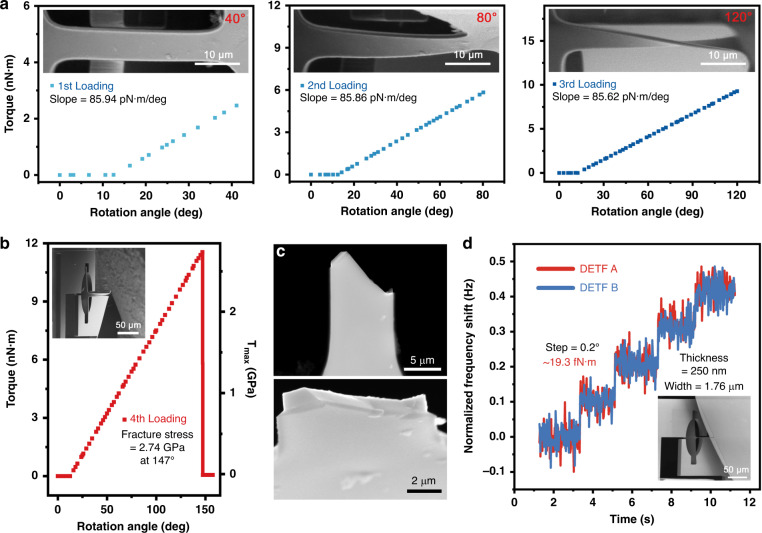
Pure torsion results of the silicon specimen. **a** Measured torque and a detailed SEM image during three loading times of the same sample under torsion angles of 40, 80, and 120 degrees. **b** In the fourth loading, the sample was fractured, and the fracture stress was calculated to be 2.66 GPa. **c** Breakage of the specimen at a torsion angle of 147.2 degrees. **d** Evaluation of the sensor resolution with a silicon dioxide nanobeam

By utilizing the method described above, torsion analysis of the specimen was achieved. To verify the feasibility and repeatability of the system, we performed four loading-unloading experiments on the same specimen. The torsion angles of the first three tests were 40, 80, and 120 degrees. When pure torsion was applied to the cross-scale specimen, real-time frequency readings of DETFs A and B were achieved at the same time, and the force and torque were precisely determined according to the calibration value. The angles versus torque measured in the three experiments are shown in Fig. 6a with different colors. Because the rectangular groove on the spline had a certain shape (width = 30 μm and length = 140 μm), when the cross-scale specimen rotated from the initial position, which was relatively parallel to the groove, the specimen did not directly touch the two finger beams in the first 12.3 degrees, and consequently, no torque was applied. After a 12.3 degree rotation, the frequency of the two DETFs increased proportionally with respect to the angle. After reaching the target position, the cross-scale specimen was reversed and removed, followed by a repeated loading test. The three torque-angle curves had nearly the same slope, which indicates that the material exhibited full elastic deformation during the tests. In the fourth test, when twisted at 147.2 degrees, the cross-scale specimen failed, as shown in Fig. 6b. The applied torque can be described by:

4 $$T = \left( {\frac{{\Delta f}}{{SF_A}} + \frac{{\Delta f}}{{SF_B}}} \right) \cdot L$$

where Δ*f*_*A*_ and Δ*f*_*B*_ are the frequency shifts of DETFs A and B, *SF*_*A*_ and *SF*_*B*_ are their corresponding scale factors, and *L* = 70 μm is the arm of the force. The top view and cross section of the fracture are shown in Fig. 6c; the other part of the specimen immediately flew away the scope after failure. When warping of the beam is ignored, the shear modulus can be obtained^36^:

5 $$G = \frac{{Tl}}{{\xi _1\varphi wt^3}} = 47.3\, {\mathrm{GPa}}$$

where *ξ*_1_ is a parameter depending on the ratio of the width and thickness; $\varphi$ is the torsion rate; and *w*, *t*, and *l* are the width, thickness and length of the beam, respectively. Using the membrane analogy^37^, the stress loaded on the rectangular cross section can be estimated:

6 $$\tau _{{\mathrm{max}}} = G\varphi _{{\mathrm{max}}}t - \frac{{8G\varphi t}}{{\pi ^2}}\mathop {\sum}\nolimits_{n = 1,3,5, \ldots }^\infty {\frac{1}{{n^2\cosh \left( {\frac{{n\pi w}}{{2t}}} \right)}}}$$

In our case $\frac{w}{t} = 6.79$, we obtain

7 $$\tau _{{{\max}}} = 0.999G\varphi _{{\mathrm{max}} }t = 2.66\,{\mathrm{GPa}}$$

Notably, the theory used to analyze the shear modulus of the specimen is crucial. The calculated shear modulus values are 46.0 GPa and 47.3 GPa according to Timoshenko theory^38^ and the Oden method, respectively. Our test results were slightly less than the shear modulus of bulk silicon 50.9 GPa^39^. The inconformity mainly comes from the manufacturing error and size measurement error of the samples. The fracture occurred when the maximum strain was 5.78% while the fracture stress was 2.66 GPa, which is 5 times stronger than its bulk counterparts^40^. Compared to silicon beams with widths of 5–75 μm^41–43^, the specimen showed approximately 2 times higher fracture strength, which coincides with the trend ‘smaller, stronger’.

We further verified the resolution of the sensor by using a silicon dioxide submicron-scale specimen (thickness = 250 nm, width = 1.76 μm). During the experiment, the specimen was twisted with a step of 0.2°. As demonstrated in Fig. 6d, a torque variation of 19.3 fN·m was easily distinguished. After analyzing the measured data during each step, a maximum standard deviation of 5.6 fN·m was obtained, close to the Allan deviation deduced theoretical resolution of the sensor of 4.78 fN·m, which indicates that fN·m torque sensing is achievable.

## Conclusions

In this paper, we demonstrated a nanorobot-aided pure torsion test system with a sophisticated microassembly scheme based on hybrid feedback. Benefiting from the high resolution and wide dynamic range of the proposed MEMS sensor, our system can perform testing experiments of various materials with different sizes from the micron to nanometer scale. The experimental results of silicon and silicon dioxide specimens indicate the high reliability and repeatability of the system. Interestingly, the silicon microbeam is 5 times stronger than its bulk counterparts, with a fracture stress of 2.66 GPa and a fracture strain of 5.78%. Our system provides a comprehensive solution for the mechanical analysis of micro/nanomaterials and will have high potential in the industrial and research fields.

## Supplementary information

Supplementary material

Assembly Video
