# Supplementary material for: Robot-aided fN∙m torque sensing within an ultrawide dynamic range
Source: Microsyst Nanoeng. 2021 Jan 4;7:2. doi: 10.1038/s41378-020-00231-0 (PMC8433428; doi:10.1038/s41378-020-00231-0)
Supplement: Supplementary file 1 — Supplementary material [file 41378_2020_231_MOESM1_ESM.docx]

**Robot-aided fN∙m torque sensing within an ultrawide dynamic range**

**Authors**

Shudong Wang,^1,2^ Xueyong Wei,^1^* Haojian Lu,^2^ Ziming Ren,^1^ Zhuangde Jiang,^1^ Juan Ren,^1,3^ Zhan Yang,^4^ Lining Sun,^4^ Wanfeng Shang,^5^ Xinyu Wu,^5^ Yajing Shen^2^*

**Affiliations**

^1^State Key Laboratory for Manufacturing Systems Engineering, Xi’an Jiaotong University, Xi’an 710049, China. *Corresponding author: [seanwei@mail.xjtu.edu.cn](mailto:seanwei@mail.xjtu.edu.cn)

^2^Mechanical and Biomedical Engineering Department, City University of Hong Kong, Hong Kong, SAR 999077, China. *Corresponding author: [yajishen@cityu.edu.hk](mailto:yajishen@cityu.edu.hk)

^3^Department of Mechatronics, Chang’an University, Xi’an 710054, China.

^4^Robotics and Microsystems Center, Soochow University, Suzhou, 215021, China.

^5^Shenzhen Institutes of Advanced Technology, Chinese Academy of Sciences, Shenzhen 518055, China.

**Supplementary Materials for**

**Robot-aided fN·m torque sensing within an ultrawide dynamic range**

**This file includes:**

**Supplementary Figures S1 to S4**


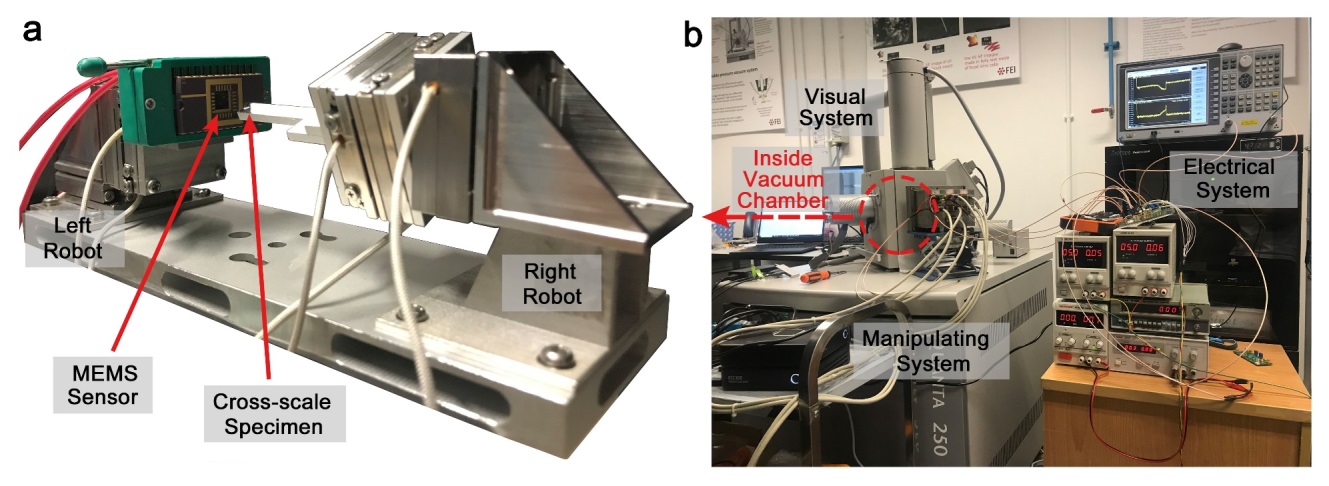


**Supplementary Figure S1**. **Diagram of the material analytics system.** The system described in this article consists of four parts: **(a)** the nanorobotics system, **(b)** the visual system, the manipulating system and the electrical system. The MEMS sensor was imbedded in a chip carrier and rigidly fixed on the left robot. The electrical signal can be transmitted through the gold bonding wires. The visual system comprises a scanning electron microscope and a computer that can record the micrographs in real time. The manipulating system includes two nanoscale-resolution controllers integrated with high-precision grating rulers. The controlling signal can be transmitted inside the scanning electron microscope through a vacuum flange. The electrical system consists of a vector network analyzer, the homemade oscillator circuits, the frequency counters, and the DC power sources. This system can monitor the frequency output of the MEMS sensor.


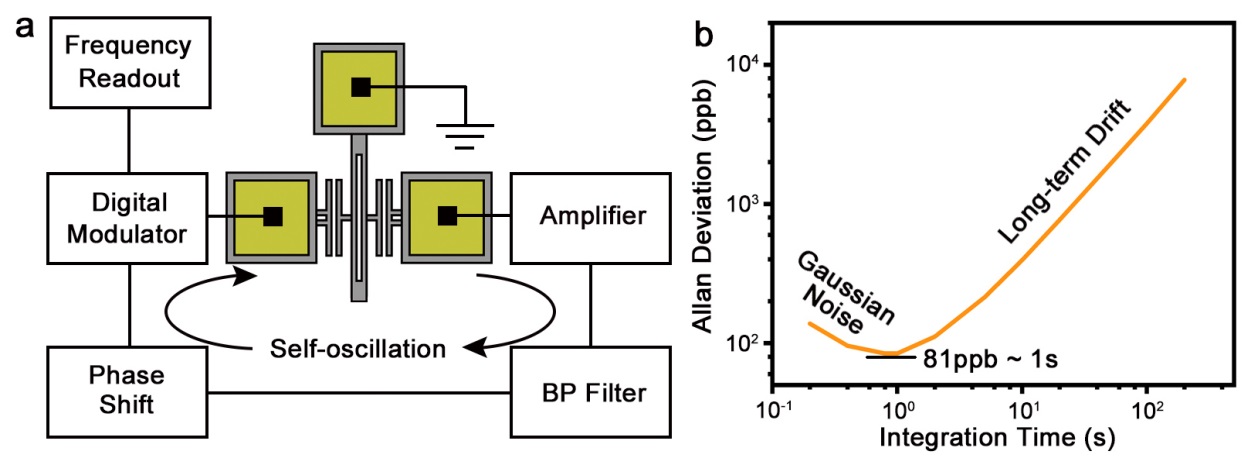


**Supplementary Figure S2. Real-time frequency readout of the oscillator.** **(a)** Schematic diagram of the homemade closed-loop oscillation circuit. This circuit consists of a transimpedance amplifier, a bandpass filter, a phase shift, a digital modulator, and a frequency readout module. When the initial condition of the self-oscillation is satisfied, i.e., the system gain was larger than 0 dB and the feedback phase was 2π, the system will self-oscillate at the same frequency as the DETF. Moreover, we can achieve real-time monitoring through the frequency readout module. **(b)** Allan deviation of the recorded frequency data when the sensor was under static conditions. As the integration time increased, the Allan deviation decreased first and then increased, whereas the minimum occurred when $\tau=1$. The theoretical resolution of the sensor can be estimated according to formula:

$$R=\frac{f_{0}\cdot\varepsilon_{Allan}}{SF}=\frac{338000Hz\times81.2ppb}{401.9Hz/\mu N}=68.3pN$$

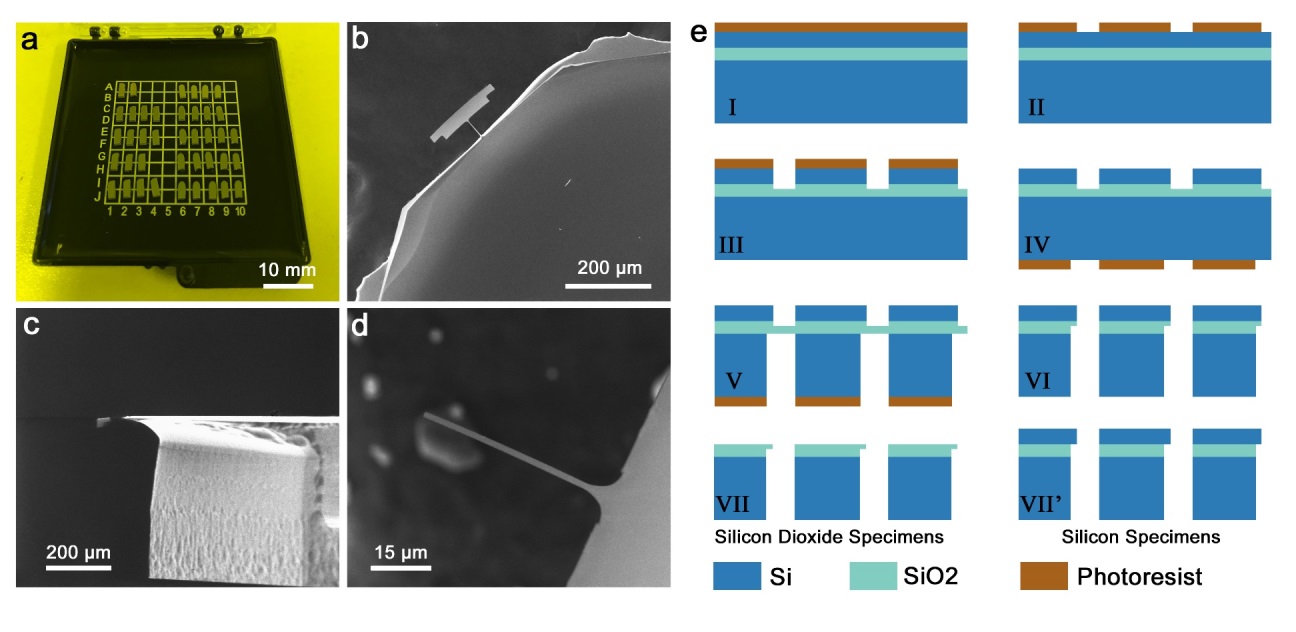


**Supplementary Figure S3. Manufacturing of the cross-scale specimen. (a)** Array of cross-scale specimens manufactured through SOI-MEMS technology. The samples of silicon and silicon dioxide can be processed through the same approach. **(b)** Top view of a silicon specimen. **(c)** Side view of a silicon specimen. **(d)** Top view of a silicon dioxide cantilever. Each specimen consists of a grip (3 mm×1.6 mm×0.5 mm) and a tip (material sample to be tested, with a minimum size of approximately 250 nm). **(e)** The processing flow used in this work, including front-side photo etching, ICP morphology etching, back-side photo etching, silicon deep etching, etc.

**­**
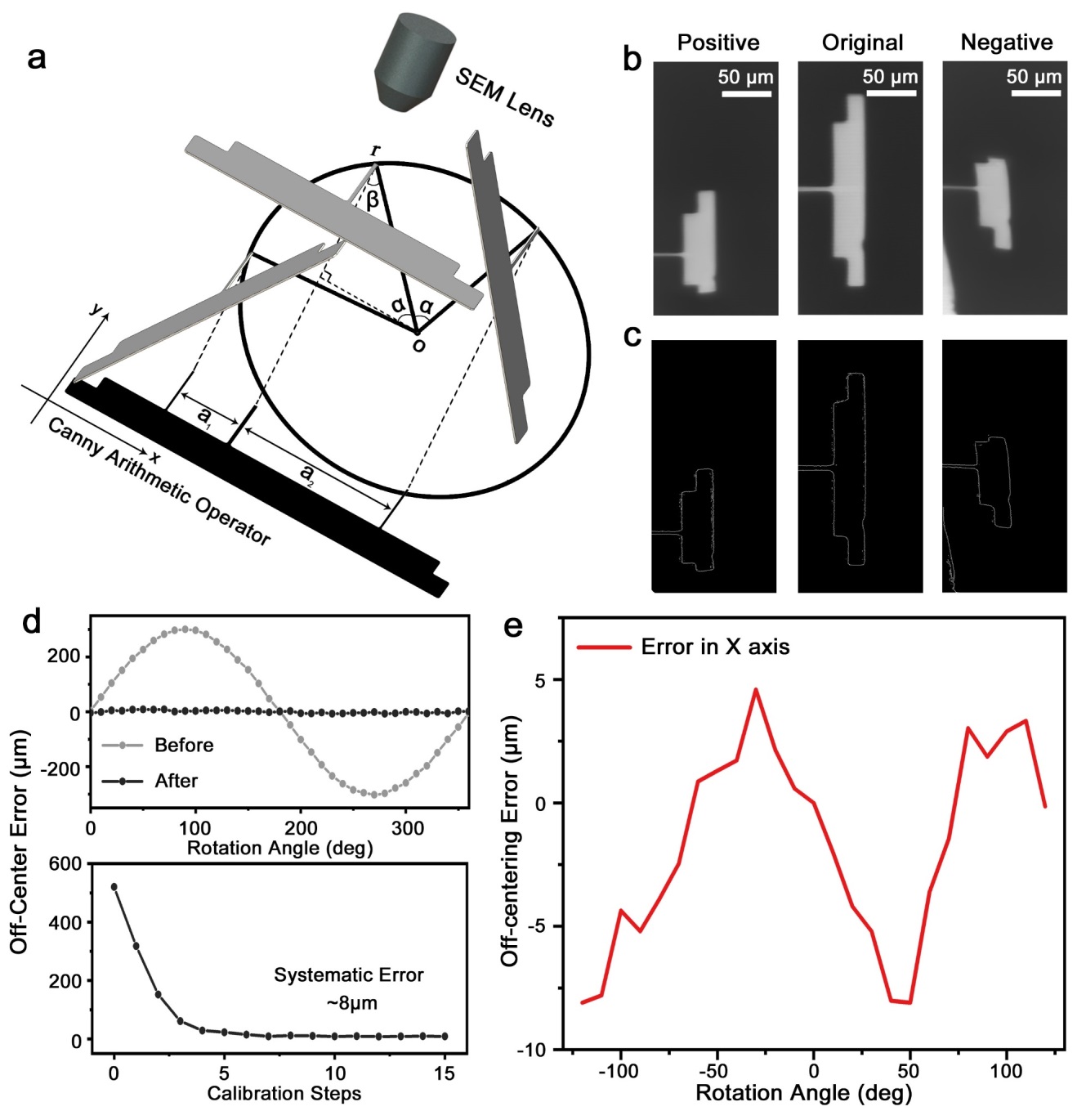
**Supplementary Figure S4. Off-center error cancellation based on three-point prealignment. (a)** Diagram of the three-point prealignment. **(b)** The specimen was rotated forward and backward by θ degrees during the alignment, and three micrographs of the relative position can be obtained. **(c)** The edges of the specimens can be extracted through the Canny operator. In the Microsoft Visual Studio environment, our system can semiautomatically calculate the eccentricity error and control the movement of the right robot to eliminate the off-center error. **(d)** Before the prealignment, the off-center error was roughly hundreds of microns. After serval semiautomatic processing steps, the error was reduced to less than 10 μm. **(e)** However, there is still an inevitable error after the alignment. There are at least three error sources: the resolution limit of the SEM image, random errors introduced by Canny operator, and inherent errors from the grating rulers and the nanopositioners.
